# Reduction of human chorionic gonadotropin beta subunit expression by modified U1 snRNA caused apoptosis in cervical cancer cells

**DOI:** 10.1186/1476-4598-7-26

**Published:** 2008-03-14

**Authors:** Anna Jankowska, Samuel I Gunderson, Miroslaw Andrusiewicz, Beata Burczynska, Anna Szczerba, Artur Jarmolowski, Ewa Nowak-Markwitz, Jerzy B Warchol

**Affiliations:** 1 Department of Cell Biology, University of Medical Sciences, Rokietnicka 5D, Poznan, Poland; 2 Department of Molecular Biology and Biochemistry, Rutgers University, Piscataway, NJ 08854, USA; 3 Department of Gene Expression, Institute of Molecular Biology and Biotechnology, Adam Mickiewicz University, Miedzychodzka 5, Poznan, Poland; 4 Division of Obstetric & Gynecology, Department of Gynecologic Oncology, University of Medical Sciences, Polna 33, Poznan, Poland

## Abstract

**Background:**

Secretion of human chorionic gonadotropin, especially its beta subunit by malignant trophoblastic tumors and varieties of tumors of different origin is now well documented; however the role of hCG in tumorogenesis is still unknown.

**Results:**

This study documents the molecular presence of human chorionic gonadotropin beta subunit in uterine cervix cancer tissues and investigates a novel technique to reduce *hCGβ *levels based on expression of a modified U1 snRNA as a method to study the hormone's role in biology of human cervical cancer cells cultured *in vitro*. The property of U1 snRNA to block the accumulation of specific RNA transcript when it binds to its donor sequence within the 3' terminal exon was used. The first 10 nucleotides of the human U1 snRNA gene, which normally binds to the 5'ss in pre-mRNA were replaced by a sequence complementary to a 10-nt segment in the terminal exon of the *hCGβ *mRNA. Three different 5' end-mutated U1 snRNA expression plasmids were tested, each targeting a different sequence in the *hCGβ *mRNA, and we found each one blocked the expression of *hCGβ *in HeLa cells, a cervix carcinoma cell line, as shown by immunohistochemistry and qRT-PCR. Reduction of *hCGβ *levels resulted in a significantly increased apoptosis rate with almost 90% of cells transfected with modified anti-*hCGβ *U1 snRNAs showing morphological changes characteristic of the apoptotic process.

**Conclusion:**

These data suggest that human chorionic gonadotropin beta subunit may act as a tumor growth-stimulating factor.

## Background

Human chorionic gonadotropin is a heterodimeric glycoprotein that belongs to the family of related hormones included FSH, LH and TSH. The hormone is composed of two non-covalently linked subunits, the common alfa subunit (hCGα) and the hormone specific beta subunit (hCGβ) [1]. Human chorionic gonadotropin (hCG) is a hormone physiologically produced by the placenta to maintain the progesterone production of corpus luteum of pregnancy, which prepares the uterus for implantation and for embryonic and placental development [2].

The ectopic production of hCG and its subunits by patients with nontrophoblastic cancers has been reported by many authors lately [3]. The free subunit was originally considered as biologically non-functional, however it was shown recently that free β-subunit may stimulate tumor growth or inhibit its apoptosis and the elevated serum level of hCGβ is frequently associated with higher aggressiveness of cancer and its resistance to therapy [4,5].

We recently reported the expression of hCGβ in patients with nontrophoblastic gynecological cancerous tissues showing that the beta subunit of human chorionic gonadotropin is produced by individual cancer cells. The distribution and the amount of hCG was heterogeneous and was not characteristic for all of the cells of the study tissue [6,7].

Here we confirm that the presence of the beta subunit of human chorionic gonadotropin's mRNA and protein is a characteristic feature of cervical carcinomas. The biological mechanisms behind the association of *hCGβ *expression in cancer cells remains unclear. Silencing gene expression provides a powerful tool for analyzing gene function, however the effectiveness of RNA inhibition strategies has been variable. Based on this we investigated a novel mRNA silencing methodology as a method to study *hCGβ *role in human cervical cancer cells cultured *in vitro*. Since nearly all mRNAs are postranscriptionally modified by 5'capping, splicing, 3' cleavage and polyadenylation it was postulated that agents interfering with processing of specific transcripts have the potential to be an effective anti-RNA strategy. Recently an approach for reducing mRNA output of a target gene was reported that is based on a modified U1 snRNA transcript as an effector. The interaction of U1 snRNP (small nuclear ribonucleoprotein particle) via base paring close to the polyadenylation signal results in inhibition of the cleavage or polyadenylation portion of the 3' end processing reaction [8,9]. This effect was applied to suppress other genes. A significant inhibition of gene expression was achieved by using a modified U1 snRNA in which the first 10 nucleotides have been substituted with a sequence complementary to the gene of interest present within the terminal exon [10-12].

In our study we exploit 5' end-mutated U1 snRNAs to block the expression of *hCGβ *in a cervix carcinoma cell line. The results have been demonstrated that U1 snRNA base paired to this target mRNA inhibits the expression of the beta subunit of human gonadotropin gene causing apoptotic death of affected cells.

## Results

### Expression of hCGβ is a characteristic feature of cervix carcinoma cancer

**Figure 1:**
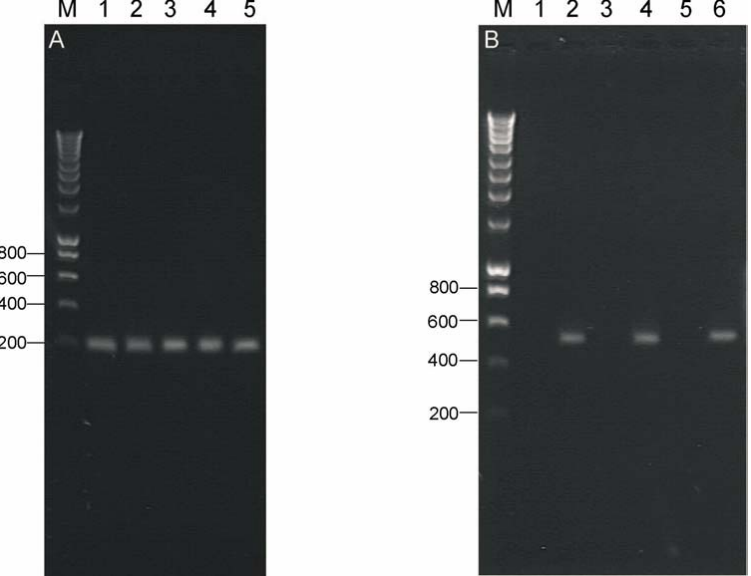
**Expression of human chorionic beta subunit in uterine cervix**. **(A) **Electrophoretic separation showing representative RT-PCR results performed for uterine cervix carcinomas. A 210 bp fragment of *hCGβ *was amplified for the uterine cervix carcinoma (lanes 1 – 4) and placenta (lane 5) samples. Molecular size marker is given in lane M. **(B) **RT-PCR analysis of total RNA from normal myometrium (lanes 1–2) and uterine cervix tissue (lanes 3–6). Lanes 1, 3 and 5 used RT-PCR primers specific to the *hCGβ *mRNA as was done in Figure 1A. Lanes 2, 4 and 6 are control RT-PCRs with primers specific to the *β-actin *mRNA (predict a 509 bp product) to confirm the integrity of the RNA. M – size marker.

To verify at the molecular level the presence of *hCGβ *in uterine cervix carcinoma, total RNA was isolated from cancer tissue of patients, reverse transcribed, and a 210 bp fragment corresponding to *hCGβ *nucleotides 348–557 [GenBank: NM_033043] was amplified. In total, 15 samples of human uterine cervix carcinoma and 4 samples of placenta were analyzed. A representative example of the RT-PCR data is shown in Figure 1A where it is evident that we were able to amplify a specific fragment of *hCGβ*. Sequencing of the amplified fragment confirmed its identity with *hCGβ*.

There was, however, no amplification of *hCGβ *fragment when cDNA synthesized from RNA isolated from tissue lacking cancerous changes (myometrium and uterine cervix) was used as a template for the PCR reaction (Figure 1B. lanes 1, 3, 5). The presence of the 509 bp fragment corresponding to *β-actin *gene nucleotides 434–942 [GenBank:X00351] in the tissues lacking *hCGβ *served as a control and confirmed the integrity of isolated RNA (Figure 1B. lanes 2, 4, 6).

We conclude that the expression of *hCGβ *is a property of uterine cervix tumor cells.

### Human chorionic gonadotropin is synthesised by cancer cells of the uterine cervix

**Figure 2:**
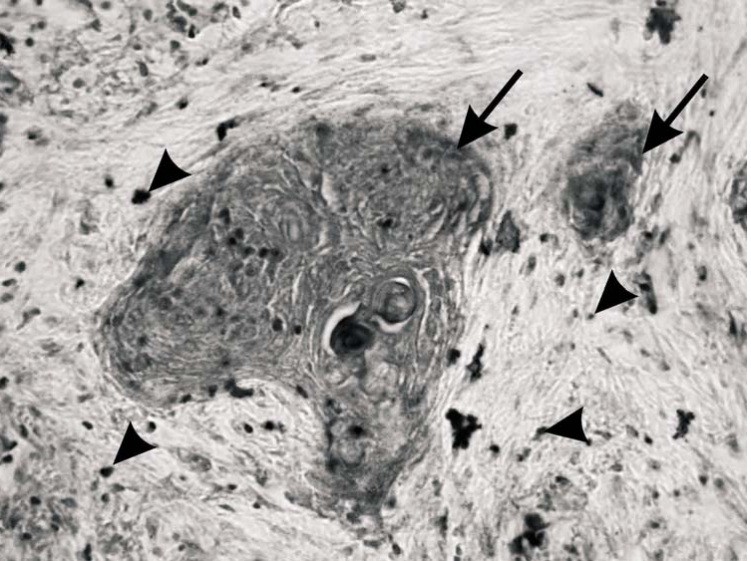
**Immunolocalisation of hCG in cervical carcinoma**. Immunohistochemistry was performed using specific antibodies against hCG on paraffin section of tumor tissue. Hormone staining was localized predominantly in the neoplastically transformed epithelial cells as indicated by the arrows. Positive immunostaining was also detected in single cells in stroma – arrowheads. Original magnification, ×400.

To verify the presence of hCG on the protein level and to address localisation of the hormone in cancer tissue, immunohistochemical analyses were carried out with primary antibodies against hCG. Hormone staining was localized predominantly in the neoplastically transformed tumor epithelial cells, characterised by uniform and homogenous cytoplasmic staining, however a single positive cells in stroma were detected (Figure 2).

No labeling was observed in the control reactions – where the primary antibodies were omitted (data not shown).

### Inhibition of hCGβ in HeLa cell line using modified U1

**Figure 3:**
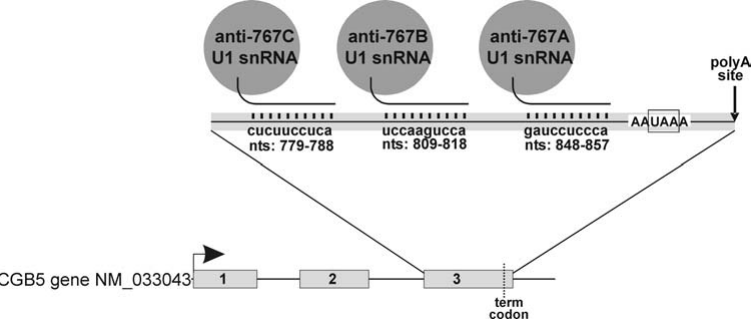
Schematic of the 3 anti-*hCGβ *U1 snRNA targeting vectors containing a U1 snRNA gene to drive expression of the anti-*hCGβ *U1 snRNAs. Schematic showing predicted base pairing interaction of each anti-*hCGβ *U1 snRNA with the *hCGβ *mRNA.

To block the expression of *hCGβ *in tumor cells the mRNA was targeted with three plasmids that encode U1 snRNA with 5'end-mutated U1 *snRNA *transgenes designed to recognize a 10 nucleotide-long sequences in the 3' terminal exon of *hCGβ *pre-mRNA (Figure 3).

**Figure 4:**
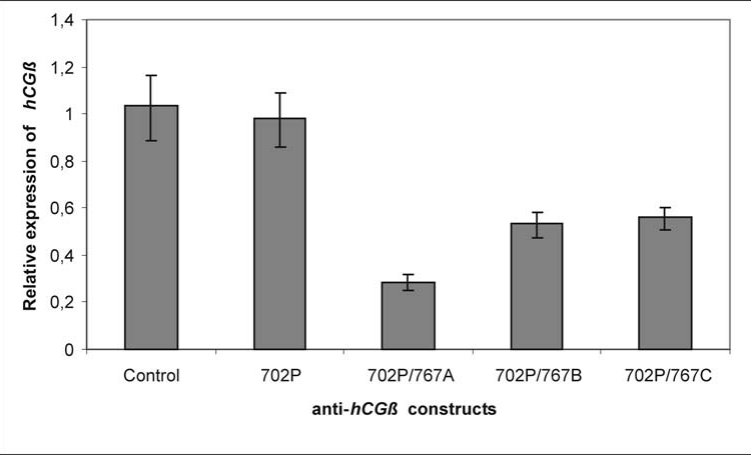
**Expression of *hCGβ *in HeLa cells transfected with anti-*hCGβ *U1 snRNA**. Total RNA was isolated from cells, and cDNA was synthesized. HeLa cells transfected with plasmids 702P/767A, 702P/767B and 702P/767C expressed decreased *hCGβ *message relative to control cells and cells transfected with control plasmid 702P. Columns – mean of triplicate experiments; bars – SE.

Because inhibition levels are limited by transfection efficiency – for example a 90% transfection rate can at most give a 10-fold inhibition [11], and in case of using HeLa cell line and lipofectamine™2000 transfection reagent the efficiency estimated by GFP reporter gene expression was lower than 30% we applied quantitative RT-PCR to show the silencing of human chorionic gonadotropin beta subunit expression. *hCGβ *transcripts were detected in both control and transfected cells however, all 3 U1 snRNA anti-*hCGβ *constructs clearly reduced the hormone's transcription as was showed by real time PCR results. The highest – 3-fold inhibition was observed for 702P/767A plasmid. Constructs 702P/767B and 702P/767C decreased *hCGβ *mRNA amount 2-times while the expression level of cells transfected with 702P plasmid and control cells was the same (Figure 4). Using FuGENE – HD Transfection Reagent we got 60% transfection efficiency and the level of hormone inhibition was the same as when transfection efficiency was 30% (data not shown).

**Figure 5:**
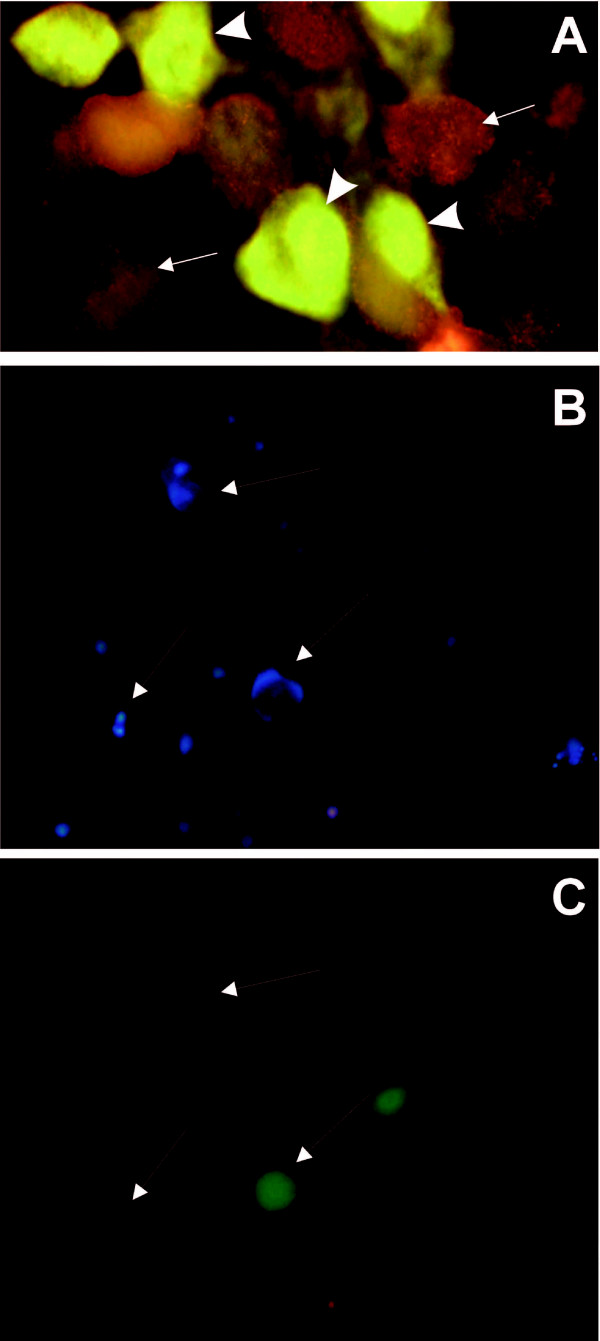
**Anti-*hCGβ *constructs knock down human chorionic gonadotropin protein expression and cause apoptosis**. **(A) **Immunohistochemistry was performed using primary antibodies against hCG and secondary antibody conjugated with Cy3. The positive staining (orange – FT 510 filter) was observed in cells lacking anti-*hCGβ *construct – arrowheads. hCG was not detected in cells transfected with the anti-*hCGβ *U1 snRNA construct 702P/767A that co-expresses GFP (green – FT 510 filter) as a marker – arrows. Original magnification ×1000.**(B) **Blue Hoechst staining of living cells. **(C) **Propidium iodide red staining of dead cells and GFP staining of cells transfected with U1 constructs. Chromatin condensation and fragmentation in the nuclei of apoptotic cells stained were showed by arrows. Original magnification, ×400.

The immunohistochemistry data confirmed the blocking of human chorionic gonadotropin synthesis. HeLa cells transfected with U1 snRNA *anti-hCGβ *constructs, and cultured for 72 hours were fixed and immunostained for hCG. We found that all three anti-*hCGβ *constructs, but not the 702P control plasmid, attenuated the human chorionic gonadotropin expression to levels below detection (Figure 5A).

### Silencing of hCGβ results in apoptosis of cancer cells

Next we determined whether the expression of 5'end-mutated U1 snRNA transgenes causing the inhibition of *hCGβ *gene can affect the viability of cells. To assess the effect of transfection of anti-*hCGβ *constructs on cancer cells the morphological features after staining with propidium iodide and Hoechst 33342 were observed. The cells transfected with a modified U1 snRNA showed changes typical for apoptosis. The nuclei condensation and fragmentation was detected particularly in approximately 90% cells expressing U1 snRNA anti-*hCGβ *construct as well as in some neighbouring cells lacking the modified U1 snRNA (Figure 5B,C). The number of these latter cells does not exceed 20%. The cells transfected with the 702P control construct did not show any significant changes and only a few apoptotic cells were observed (data not shown).

**Figure 6:**
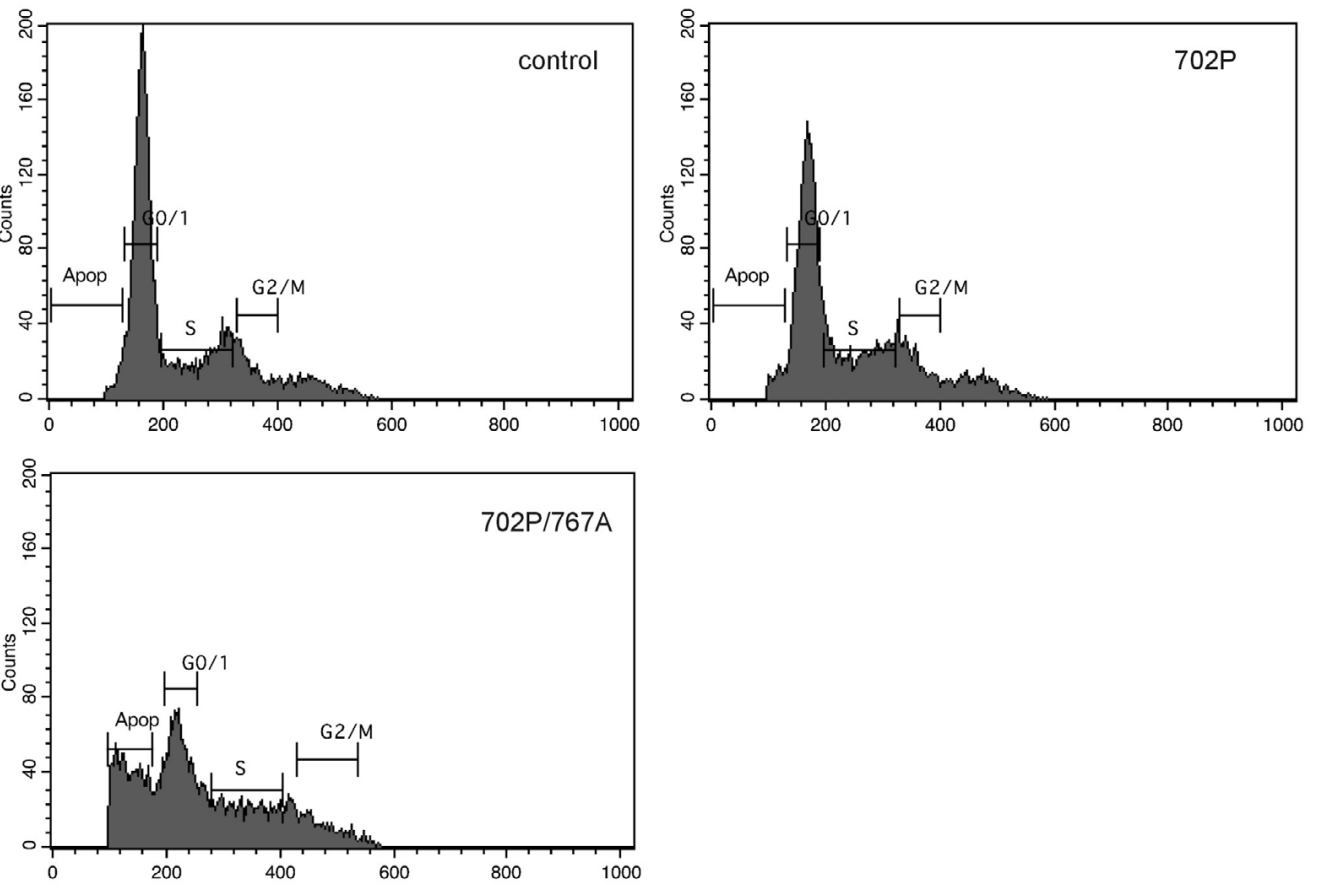
**Effect of transfection with anti-*hCGβ *U1 snRNA on apoptotic rate in HeLa cells**. FACS analyses using propidium iodide staining was performed for DNA content measurement. Apoptotic population was measured as the percentage of total cell population with sub-G_1 _DNA content. Results shown represents experiments performed for control cells and cells transfected with plasmid 702P and 702P/767A, respectively.

Changes in cell cycles distribution were monitored by flow cytometry. The inhibition of *hCGβ *expression by anti-*hCGβ *constructs result in increase in distribution of cells at sub-G_1 _peak – apoptosis peak (Figure 6). The percentage of apoptotic cells was increased to 26.65%, 23.59% and 22.48% for 702P/767A, 702P/767B and 702P/767C plasmids respectively (Table 1).

**Table 1:** Effect of *hCGβ *silencing on cell cycle of HeLa cells. n = 3. Mean ± SD.

	**Apoptotic [%]**	**G0/1 [%]**	**S [%]**	**G2/M [%]**
**Control**	1.82 ± 0.09	56.06 ± 2.80	23.38 ± 1.17	8.01 ± 0.40
**p702P**	3.13 ± 0.16	45.73 ± 2.29	26.43 ± 1.32	11.33 ± 0.57
**p702P/767A**	26.65 ± 1.33	27.15 ± 1.36	21.36 ± 1.07	8.56 ± 0.43
**p702P/767B**	23.59 ± 1.18	31.27 ± 1.56	19.24 ± 0.96	15.29 ± 0.76
**p702P/767C**	22.48 ± 1.12	30.76 ± 1.54	22.27 ± 1.11	12.96 ± 0.65

## Discussion

Human chorionic gonadotropin (hCG), a heterodimeric sialoglycoprotein hormone composed of two noncovalently linked subunits – α (hCGα) and β (hCGβ) is physiologically produced by the placenta [13]. Numerous studies demonstrated that besides placenta and malignant trophoblastic disease, varieties of tumors of different origin secreted hCG and especially its β-subunit [3,14,15]. As previously demonstrated by Acevedo and Hartsock, in a nude mouse model, high expression of membrane-associated hCGβ correlates with primary malignant growth and metastatic phenotype of cancer [14].

The findings of present study indicate the *hCGβ *gene is active in uterine cervix cancers. In all 15 specimens of the cervical carcinomas that were analyzed, the transcripts of *hCGβ *were present. Even in slightly advanced carcinomas such as carcinoma of uterine cervix, FIGO stage 0, IA we found the mRNA of *hCGβ*. At the same time we showed that tissue lacking the cancerous changes did not express *hCGβ *mRNA. The RT-PCR results were proofed by immunohistochemical analysis, which revealed that hCG is expressed in cancer cells within a cervical carcinoma. Thus, the expression of *hCGβ *is a characteristic feature of cervical tumor tissue, however the role of hCG in tumorogenesis is unknown.

The discovery of the crystal structure of hCG demonstrated that dimeric hCG forms the cystine knot structure [16]. The structure of hCG resembles the structure of known growth factors, namely TGFβ (Transforming Growth Factor), NGF (Nerve Growth Factor) and PDGFβ (Platelet Derived Growth factor) [16,17]. It was actually shown that subjection of an hCGβ-secreting bladder tumor cell line to stimulation by hCGβ *in vitro* led to an increase in cell number, in a dose dependent manner [4].

Guided by these observations we reasoned that the reduction of the hormone level would result in decrease of cell viability. We used a new strategy to knock down the expression of *hCGβ *in cultured cervix carcinoma cells. Mammalian U1 snRNP contains ten proteins bound to U1 snRNA and binds to the exon-intron boundary of pre-mRNA to direct early steps of splicosome formation [18]. Binding involves the interaction between nucleotides 2–11 of the human U1 snRNA and the 5' splice site of pre-mRNA. A modified U1 snRNA can be used as an alternative to siRNA, antisense RNA or ribozyme approach for reducing the mRNA output of a target gene. In our study we examined whether binding of modified U1 snRNAs to 10 nucleotide regions within the terminal exon of human chorionic gonadotropin beta subunit was able to inhibit the hormone synthesis.

We demonstrated that U1 complementary to three different target regions of *hCGβ *pre-mRNA results in suppression of the hormone expression both on the mRNA and protein level. The fact that three different modified U1 snRNAs gave similar results would argue that *hCGβ *reduction, as opposed to off-target effects by each modified U1 snRNA, is responsible for the effects we observe. Our results indicated that HeLa cells trasfected with U1 snRNA anti-*hCGβ *constructs showed 2–3-fold inhibition of human chorionic gonadotropin beta subunit transcripts. In cancer cells expressing the modified U1 snRNAs hCG protein was undetectable. Further analysis showed that knocking down gonadotropin expression significantly increases the apoptosis rate what was shown by flow cytometry and morphological changes characteristic for apoptotic process. Thus, the inhibition of the beta subunit of human chorionic gonadotropin expression leads to induction of programmed cell death.

It was already shown that the addition of anti-hCGβ antibodies to the culture media of bladder cancer cell line inhibit the growth of the cells which produce endogenous free hCGβ [4]. Furthermore Rivera et al., as a result of applying the antisense RNA specific to *hCGβ*, demonstrated the loss of tumorogenic potential of human lung cancer cells (ChaGo cells), cultured *in vitro *[19]. Also the transfection of an antisense chorionic gonadotropin beta gene into choriocarcinoma cells suppressed cell proliferation and increased the apoptosis-positive rate of JAr cells [20].

Recent reports suggest that free hCGβ is acting as a growth-stimulating factor, but the population growth is not the result of increased replication rate but of reduction in cell apoptosis [3,21]. Since the induction of epithelial cell apoptosis is a well-established action of TGFβ and the topological homology of hCGβ and TGFβ was previously shown it was suggested that the anti-apoptotic effect of hCGβ could potentially occur by blocking the TGFβ receptor. Blocking of the receptor by free hCGβ could prevent any further interactions with other components that are necessary for initiating cytoplasmic signaling that leads to apoptosis [3].

The inhibition of *hCGβ *expression by modified U1 snRNAs caused apoptosis both in cells expressing U1 *anti-hCGβ *construct as well as in neighbouring cells, lacking the modified U1 snRNA. This neighboring cell effect could be explained by a decreased level of hCGβ in culture medium, which allows the interactions between the receptor and ligands essential for induction of apoptosis. Therefore our results are in good agreement with the date published previously.

## Conclusion

The results of the study demonstrated that blocking of human chorionic gonadotropin beta subunit by modified U1 complementary to three different target regions of its pre-mRNA results in suppression of the hormone expression both on the mRNA and protein level. The knocking down gonadotropin expression significantly increases the apoptosis rate of affected cells.

While current anti-hCGβ therapeutic strategies are based on antibody blockade of ectopically expressed hCGβ here we demonstrated, *in vitro*, the potential of mRNA silencing gene technology to achieve the same therapeutic results via the same molecular mechanism, increased endogenous apoptosis.

## Materials and methods

The specimens of gynecological cancer tissue were obtained from patients treated by surgery at the Department of Gynecologic Oncology, Poznan University of Medical Sciences in 2004 – 2006. The uterine cervix carcinoma group consisted of 15 patients with epithelial carcinoma of the uterine cervix. In all patients, histological proof, including tumor grading, was obtained and the staging was performed according to FIGO.

Histology groups were as follows: Carcinoma planoepitheliale (12 cases; tumor grading: G1 – n = 6, G2 – n = 6; FIGO: IA – n = 2, IIA – n = 1, IB – n = 7, IIB – n = 1, IIIAB – n = 1), Glassy cell carcinoma (1 case; tumor grading – not determinate; FIGO IB), Basaloid cell carcinoma (1 case; tumor grading – not determinate; FIGO IIB), Carcinoma intraepitheliale CIN III (1 case; tumor grading – not determinate; FIGO 0). No patients received chemotherapy or radiotherapy prior to operation.

The control included two samples of the myometrium and three samples of the uterine cervix that lacked cancerous changes as estimated by a pathologist's macroscopic and microscopic examination. Four placentas from term pregnancies served as a control. The placentas were obtained from the Department of Perinatology Karol Marcinkowski University of Medical Sciences, Poznan, Poland.

Tissue samples were frozen in liquid nitrogen or fixed in 4% paraformaldehyde.

The study was approved by the ethics review board of Poznan University of Medical Sciences and all patients participated after informed consent.

HeLa cell line, established from cells of cervix carcinoma and producing human chorionic gonadotropin served as an *in vitro* model of nontrophoblastic gynecological cancer. The cells were cultured and passaged under standard conditions.

### RNA isolation and RT-PCR

Total cellular RNA was extracted in TriPure (Roche Diagnostic, Mannheim, Germany) according to manufacture protocols.

1 μg of RNA (DNase treated) was employed individually for one reverse transcription reaction with universal primer p(dT)_10 _and Expand Reverse Transcriptase (Roche Diagnostic).

A 210 bp fragment of *hCGβ *was amplified from cDNA using the following primers: sense 5'-GCAGGGGACGCACCAAGGA-3' (nucleotides 348–367, according to cDNA sequence) and antisense 5'-CACGCGGGTCATGGTGGG-3' (complementary to nucleotides 539–557, [GenBank:NM_033043]). The amplification was performed in a reaction mixture containing: 1× Taq DNA polymerase buffer, 2.5 mM MgCl_2_, 0.2 mM dNTPs, 0.25 μM of each primer and 1 unit of BioX-Act DNA polymerase (Bioline, London, UK) with thermal profile as followed: 5 min at 95°C, 1 min at 95°C, 45 sec at 64°C, 45 sec at 72°C for 30 cycles. The quality of RNA was checked by examining ribosomal RNA bands after agarose gel electrophoresis and by amplifying *β-actin *as a control. All products were sequenced to confirm their identity.

### Quantitative RT-PCR

To quantify the amount of *hCGβ *and *GAPDH *(internal control) in HeLa cells after RNA isolation, reverse transcription real-time PCR with LightCycler 2.0 (Roche Diagnostics) and LightCycler FastStart DNA Master SYBR Green I Kit (Roche Diagnostics) was performed. 20 μl of PCR reaction mixture contain: 2.5 mM of MgCl_2_, 2 μl of SYBR Green 1 mix, 2 μl of cDNA and a 0.5 μM of each primer. *hCGβ *primers sequence was as described above and *GAPDH *primers were designed as follow: sense – 5'-CAG CCT CAA GAT CAT CAG C-3' and antisense – 5'-GAC TGT GGT CAT GAG TCC TCC-3' [GenBank:NM_002046]. The amplification program consisted of 1 cycle of 95°C with a 10 min hold, followed by 45 cycles of 95°C with a 10 s hold, annealing temperature at 62°C (*hCGβ*) or 58°C (*GAPDH*) with a 5 s hold, and 72°C with a 9 s hold. An additional step was added for fluorescence data acquisition at an elevated temperature for *hCGβ *amplification, which is 88°C with a 2 s hold. This was followed by melting curve analysis, which ran for 1 cycle at 95°C with a 0 s hold, 65°C with a 15 s hold, and 95°C with a 0 s hold at the step acquisition mode.

All experiments were performed in triplicates. PCR efficiencies were calculated from the standard curves (generated using serial dilutions of *in vitro* generated transcripts) for *hCGβ *and *GAPDH*. Melting curve analysis (LighCycler software package) was applied to ensure the specificity of the PCR reaction. A relative expression level of *hCGβ *gene was normalized with *GAPDH*.

### Immunohistohchemistry

Paraffin sections of analysed tissue fixed in 4% paraformaldehyde were used for immunohistochemical detection of hCG. Antigens were retrieved by microwave activation in citrate buffer (10 mM, pH 6.0). After being blocked in blocking buffer – TBS, pH 7.5, containing (100 mM TRIS-HCl, 0.9% NaCl, 0.05% Tween-20 (TBS-T) and 1% BSA) sections were incubated with primary rabbit polyclonal antibodies against hCG (DAKO A/S, Glostrup, Denmark) diluted 1:200 in blocking buffer for 60 minutes at 37°C in a humidified chamber and washed 4 × 15 minutes in TBS-T. AP-conjugated anti-rabbit IgG, diluted 1:200 (Sigma-Aldrich, Saint Louis, Mi, USA) and NBT/BCIP (Sigma) as the substrate were used for detection. Incubation and washing conditions were as described for primary antibodies. Controls included detection reactions carried out under identical conditions except that the primary antibodies were replaced by nonimmune serum.

At 72 hours after transfection HeLa cells were rinsed in PBS, fixed in 4% paraformaldehyde for 5 minutes at room temperature and blocked in blocking buffer. Then they were incubated with primary rabbit polyclonal antibodies against hCG diluted 1:200 in blocking buffer for 60 minutes at 37°C. For the detection of antigen-primary antibody complex the secondary Cy3-conjugated antibodies (Sigma) diluted 1:200 were used. The reaction was visualized with fluorescence microscope (Zeiss, Axioskop 2) with appropriate filters for GFP (Zeiss, FT 510) and Cy3 (BP365).

### U1 targeting constructs

3 modified U1 snRNA anti-*hCGβ *constructs designated 702P/767A, 702P/767B and 702P/767C were created by PCR-directed mutagenesis of the 5' sequence between bases +2 to +11 of a U1 snRNA expression plasmid following previously described methods (9, 10). Bases +2 to +11 of U1 snRNA normally complement the 5' splice donor but when mutated, as in these anti-*hCGβ *constructs, result in a U1 snRNA able to basepair to a target sequence in the 3' terminal exon of the human *hCGβ *pre-mRNA.

Based on numbering from the *hCGβ *[GenBank:NM_033043], plasmid 702P/767A targets nts 848–857, plasmid 702P/767B targets nts 809–818 and plasmid 702P/767C targets nts 779–788. Construct 702P is a control that expresses wild type U1 snRNA and so should not target *hCGβ*. All constructs express GFP off a separate promoter that serves as a marker for transfected cells. DNA sequencing was performed to confirm that the mutations were successfully introduced into the 5' end of the U1 snRNA expression plasmids.

### Transfection

HeLa cells were seeded so as to grow to 70–80% confluence on the day of transfection. Transfection with anti-*hCGβ *constructs utilising lipofectamine™2000 (Invitrogene, Carsbad, CA, USA) and FuGENE – HD Transfection Reagent (Roche Daignostics) was done according to the manufacturer's protocol. The analyses were performed on cells harvested 72 h following transfection. Reduction of *hCGβ *expression levels by the anti-*hCGβ *constructs were calculated after normalization to the control 702P construct.

### Apoptosis

The cells transfected with *anti-hCGβ *and 702P control constructs were stained with propidium iodide [0.1 g/ml] and Hoechst 33342 [0.125 g/ml] (Sigma) 72 hrs after transfection and chromatin changes typical for apoptosis was analysed with fluorescence microscope (Nikon Eclipse TE 200).

### Cell cycle analyses

The cells were harvested after treatment fixed in cold 70% ethanol. Fixed cells were subsequently washed and treated with 100 μg/ml RNAse A, and stained with 50 μg/ml propidium iodide. Analyses were performed with BD Facs Calibur. Three parallel samples were measured and no less than 10 000 cells were tested in one sample. Cell cycle distributions were analyses by BD Cell Quest™ Pro ver.5.2.1 software.

## Competing interests

The author(s) declare that they have no competing interests.
